# Understanding Health Research Ethics in Nepal

**DOI:** 10.1111/dewb.12109

**Published:** 2016-02-03

**Authors:** Jeevan Raj Sharma, Rekha Khatri, Ian Harper

**Keywords:** bioethics, developing world, global South, research ethics

## Abstract

Unlike other countries in South Asia, in Nepal research in the health sector has a relatively recent history. Most health research activities in the country are sponsored by international collaborative assemblages of aid agencies and universities. Data from Nepal Health Research Council shows that, officially, 1,212 health research activities have been carried out between 1991 and 2014. These range from addressing immediate health problems at the country level through operational research, to evaluations and programmatic interventions that are aimed at generating evidence, to more systematic research activities that inform global scientific and policy debates. Established in 1991, the Ethical Review Board of the Nepal Health Research Council (NHRC) is the central body that has the formal regulating authority of all the health research activities in country, granted through an act of parliament. Based on research conducted between 2010 and 2013, and a workshop on research ethics that the authors conducted in July 2012 in Nepal as a part of the on‐going research, this article highlights the emerging regulatory and ethical fields in this low‐income country that has witnessed these increased health research activities. Issues arising reflect this particular political economy of research (what constitutes health research, where resources come from, who defines the research agenda, culture of contract research, costs of review, developing Nepal's research capacity, through to the politics of publication of data/findings) and includes questions to emerging regulatory and ethical frameworks.

## Introduction

There is much recent debate around health sector research on the relationship between types of research, the nature of evidence generated, and their suitability for informing policy^1^ and to the limits of systematic reviews in the complex context of health interventions in low‐income countries.^2^ Much of the scholarship focusing on health sector research in low‐income countries has focused on pharmaceutical development and direct research on human subjects within the field of clinical trials and health experimentation.^3^ Existing debate on health research ethics tends to emphasise informed consent at the expense of the broader aspects of our ethical responsibilities.^4^

Although there has been increasing research activity around health service research and operational research in low and middle‐income countries, much less work has been done to discuss ethical dimensions of this field.^5^ The application of protocols and techniques for research into human subjects is now increasingly criticized as inappropriate or inadequate for research into more complex health systems related issues in low‐income countries.^6^ Low‐income countries are heavily dependent on external assistance to support their health systems and service delivery work. Health sector research, often organized as externally assisted interventions that generate evidence, plays a key role in shaping national policies, plans and programmes. The political economy of research in low‐income countries–including the uneven distribution of resources, external dependence on funding and limited local capacity–poses specific challenges to low‐income countries that have emerging regulatory and ethical frameworks.

Drawing on findings from our “Biomedical and Health Experimentation in South Asia” [BHESA] research project conducted in Nepal between 2010 and 2013, this article discusses the emerging regulatory and ethical fields, showcasing a low‐income country that has seen increased health research activities that range from health experiments, randomised controlled trials to research activities around health sector programmatic interventions. We began by mapping all of the experimental research ongoing in the health sector in Nepal between 2000 and 2010. The major source for our mapping was a bibliography prepared by the Nepal Health Research Council of the research studies that were approved from 2003 to 2009. In addition to the bibliography, the US registry (clinicaltrials.gov), Current Controlled Trials, and a web search of sponsors, research organizations and journal articles also served as sources of data for our mapping exercise. This provided us with an overview of the nature of health research activities in the country more broadly and health experimentation specifically. In addition, as a component of broader ethnographic research, we carried out 73 key interviews with investigators, managers and regulators soliciting their experiences and perceptions on the practice of research, the general health research environment and emerging ethical issues in the country.

One of the key themes that emerged during our research related to whether the research designed around programmatic interventions–such as feasibility studies, service delivery research, assessments and evaluations that are not seen as “health research experiments”– should be counted broadly as “health research” at all. To explore this further, and as part of the iterative nature of our research, we convened and conducted a workshop to look into the issues around the conduct of ethical review of research in the health sector in the context of Nepal. The main objective of the workshop was to bring together investigators and research managers from key institutions (academic, NGOs, government and private sector) involved in conducting health research to promote a dialogue on the current state of formal ethical review and practice in country. In short, the workshop offered an opportunity to discuss and debate the experiences and challenges regarding ethical process in the conduct of experimental research studies.^7^ This article discusses issues arising that reflect a particular political economy of research ‐ where funding comes from, who defines the research agenda, the costs of review, developing Nepal's research capacity, through to the politics of publication of research findings ‐ and includes questions relevant to emerging regulatory and ethical frameworks.

## Political economy of health sector research in Nepal

Unlike other countries in South Asia, in Nepal scientific research has a relatively recent history.^8^ Health research in Nepal has its origin in aid from the United States started in 1951. In its mission to support Nepal's development, one of the first challenges USOM (United States Operations Mission, later renamed as USAID) faced was the lack of data. A USOM document published in 1958 stated: “Reliable health statistics do not exist. This makes the assessment of health conditions and their exact nature and scope very difficult to relate to the specific problem of resource development and utilization”^9^. USOM undertook Nepal's first systematic research activity, a survey on malaria, in 1952, designed within the context of the country's Malaria control interventions. The first Nepal Health Survey was carried out in 1965‐66 with the support of the University of Hawaii and the Thomas A. Dooley Foundation. It had a budget of 250,000 USD. Its main objective was to supply baseline quantitative data to assist the Ministry of Health in planning and to be useful in measuring future progress of health work in Nepal.^10^ In its early days, “To improve project design and implementation, USAID increasingly relied on sector assessments, pilot studies, and the hands‐on field presence of Peace Corps Volunteers (PVCs).”^11^ The health development anthropologist Justice, writing of the 1970s, suggested “government and donor agencies produce many kinds of reports, often voluminous: background papers, feasibility studies, annual reports, progress evaluation and project proposals.”^12^

Over the last 60 years, health related research conducted in Nepal has been varied. It ranges across more pure biomedical research, into arenas where evidence is being generated for programmatic interventions into the health field more generally. This generation of evidence around programmatic interventions has been sustained by assemblages of local and international organizations and universities, and supported and funded by aid institutions.^13^ These assemblages and institutional forms are not only critical in the generation of evidence but also provide much‐needed networks of support for the successful up scaling of pilot projects. It is, however, impossible to trace all the research activities in Nepal, especially those that are designed to generate evidence around programmatic interventions. This is mainly because not all research activities are registered with Nepal Health Research Council (NHRC), there is no standard database and no clear definition as to what is regarded as health research. Without knowing how many and what research activities are taking place in the health sector, however defined, NHRC faces a major challenge in the regulation of research activities. There are, however, several examples of interventions that are at the interface of health research and programmatic intervention in Nepal. Here we consider the following as illustrations of these forms.

From 1988‐90, USAID funded the Nepal Nutrition Intervention Project‐Sarlahi (NNIPS) on Vitamin A capsule distribution in Sarlahi district, which was implemented by Johns Hopkins University in collaboration with the National Society for the Prevention of Blindness, Kathmandu. The project carried out a randomised, double‐masked, placebo‐controlled community trial of 28,630 children aged 6‐72 months in rural Nepal. The study results showed 30% reduction in infant mortality.^14^ Results from this study and another study funded by USAID on Vitamin A^15^ were instrumental in the government of Nepal introducing the National Vitamin A Program in Nepal.^16^ Likewise, another USAID funded ARI Intervention Trial in Jumla district was implemented by John Snow Inc. in collaboration with the Ministry of Health. This demonstrated that community health workers, with limited training, could reliably manage childhood pneumonia, a major cause of deaths in children under five years old.^17^ The positive results led the Government of Nepal–with support–to introduce a community‐based component within the national ARI control programme, and to further mobilise Female Community Health Volunteers (FCHVs) to administer antibiotics. A technical working group that consisted of the government staff, UNICEF, WHO, USAID and John Snow Inc. was established in 1993 to take the programme further.^18^

Do such programmes constitute health research in the biomedical sense, or are they more broadly development interventions? What are the broader ethical issues involved in such research activities when they are embedded within such development interventions?

A mapping exercise on health research experiments we conducted in 2011‐2012 showed that there were 162 studies undertaken in Nepal between 2000 and 2010; many other studies were undertaken in this period that did not fit the criteria of health experiments.^19^ The number of Principal Investigators (PIs) in these studies was 132. Of these 69 were Nepali, 63 were foreign, 84 were male and 48 were female. Among the 132 PIs, 63 were affiliated with universities and academic institutions, 25 with other health facilities (hospitals, clinics), 12 with INGOs, 10 with NGOs and 7 with government departments. The affiliation of 15 PIs could not be identified because the organization details were not available. A total of 26 sponsors/funders were identified, ranging from bilateral and multilateral institutions to universities and private foundations. There were 56 organizations implementing the 132 studies: 36 were local and 20 were international. The international research implementing organizations were mostly universities from the US, Belgium, Australia, UK, Japan, and Denmark and some INGOs working in Nepal, such as Care International and Heifer International. The local research organizations were NGOs or hospitals. With the exception of GlaxoSmithKline, who sponsored the trial of a Hepatitis E vaccine^20^, no other pharmaceutical companies funded trials in Nepal. The largest sector in Nepali health research was maternal and child health. There were also studies on high altitude medicine, tuberculosis, abortion, encephalitis, malaria, leprosy, typhoid and HIV, among others.

Broadly, and inductively, our research teased out the following issues and themes that were emergent in this field of health research in Nepal and their ethical implications.

*Firstly*, there has been a steady increase in the number of research activities and this is linked to a number of factors: the rise in the number of medical schools^21^ and associated research activities, which while increasing in number, are often poorly supported in terms of an institutional culture of research; and that there has been an increased demand to generate “evidence” around health sector programmatic interventions. When compared to other parts of South Asia^22^, there is relatively little biomedical research conducted in Nepal, which is limited to a few public hospitals such as Patan Hospital, Anandaban Hospital, the Teaching Hospital and a couple of private research organizations that have been established in the last 10 years. Most research activities are organised as international collaborative assemblages of aid agencies, universities, contract research organisations, NGOs, local research firms and individual researchers/investigators.

*Second*, except for a few medical education institutions, several NGOs and a few private research firms specializing in health systems research have emerged in the country that mainly work on short‐term sub‐contractual agreements with the government, bilateral, multilateral and private philanthropic organisations. The most active local research institutions include New Era, Valley Research Group, Centre for Research on Environment Health and Population, Health Research and Social Development Forum and Nepal Public Health Foundation. New Era, for example, was established by three Peace Corps Volunteers in 1971, and was the first research firm established to work in Nepal. Although it was initially registered in the United States, in 1977 it was registered in Nepal and Nepali researchers took over from the Peace Corps Volunteers. This institution is the most contracted research firm in Nepal for quantitative surveys and has worked very closely with USAID for carrying out the Demographic and Health Surveys. Given the small number of these institutions in the country, which reflects the limited research, they are often oversubscribed by the sponsors. The short‐term nature of the contracts mean that they are constantly busy in simultaneously handling multiple projects while moving to the next ones.

*Third*, a particular culture of contract research, especially within health systems research, is prevalent. There is the widespread practice of individual researchers and institutions working on short‐term contracts for sponsors, mainly INGOs, bilateral and multilateral organisations, where they work to carry out feasibility studies, evaluation studies, baseline and end‐line surveys. Generally, commissioning sponsors give preliminary research designs and have short time frames with very little or no protected time to write for publications. In some cases, they also determine the sample size and the study sites. In addition to the design, sponsors are often involved in monitoring as well as the final publication of the results. Often sponsors withheld the ownership of the data and sub‐contractors may have limited ownership on the data. The research reports do not always include the name of the researchers as authors on the front pages, but they are included in the acknowledgements section. Although contracts are formally awarded on a competitive basis, “trust” between sponsors and the contract research organisations is often rooted in prior working relationships and is the determining factor in awarding contracts. New Era, for example, continues to conduct the USAID funded Demographic and Health Surveys. For the conduct of the research itself, the types of personal and institutional networks developed during the research process are crucial to the formation and development of the research capacity. In addition, the dissemination of findings and the policy domain into which research was designed to fit is also dependent on these personalised networks. This is illustrated, for example, in an operational study on the feasibility of the distribution of misoprostol (known as *matri surakschya chakki* in Nepali) by Female Community Health Volunteers (FCHVs) in Banke district conducted between 2005‐07 under the USAID funded Nepal Family Health Programme (NFHP). The NFHP was a consortium led by John Snow Inc with a number of national and international partners. One of NFHP's focus was to tackle post‐partum hemorrhage and prevent maternal deaths in home deliveries in Nepal. Much resources and energy were expended into facilitating government involvement in all stages of the programme leading up to the successful scaling up of the intervention in national policy in 2010. Interacting, spending time and engaging with senior government officials through the programme structure opened a pathway for influencing government policy.

*Fourth*, limited locally available research funding means that local researchers and institutions actively look to engage in research collaborations with Northern research partners that include international research institutions and universities. These research partnerships and collaborations are shaped by the availability of resources and partners, and the practicalities of carrying out research as well as by the existence of networks. While there was a widespread sense that local research institutions and investigators are now involved as more active research partners, rather than being treated as ‘sleeping partners or Sherpas’, Northern partners continue to take the lead in identifying research problems, crafting research questions and drafting methodologies to organise the overall research project. Though informed and involved in the design of the protocols, lack of sufficient information on scientific debates means that the contribution of Nepal based researchers and research organisations is often limited to providing minimum feedback on the protocols.

*Fifth*, there are continuing issues with the development of research capacity within Nepal, and institutional capacity to do research related work. One the first issues is the rapid turnover of staff within these organizations. The political economy of development work in Nepal has resulted in a highly stratified pay structure. Individuals with particular skills (for example, Nepal has a severe shortage of qualified experienced statisticians) are able to move across institutional spaces and demand higher wages. This is particularly so in the Kathmandu valley where most of these organizations are based and it has resulted in stiff competition for the employment of staff with the capacity to undertake both programmatic and research based interventions. The consequent rapid turnover of staff and volatile pay structure means that institutional capacity is often flagging. In addition, in the government run institutions there is overt political interference with staff being replaced with changes in government, and where research is being conducted, direct interference with the research itself, something that the Maoists during the insurgency were particularly prone to do. The sustained political instabilities are thus a particular issue. This is as evident in the NHRC, as elsewhere, where, for example, changes in which party controls the Ministry of Health results in sweeping changes in the staffing of bureaucracies associated with the ministry.

*Finally*, as in other low‐income countries, health research in Nepal is conducted in an arena of structural inequalities where there is a lack of technology, infrastructure and funding. Laboratories, for example, do not have the most up to date equipment. Procuring this is frequently dependent on grants from external agencies, and many sponsors will not fund these overheads. Even when there is the requisite equipment, there is the broader issue of local constraints; unavailability of reagents in local markets, fuel shortages, lengthy and sustained power cuts throughout the year, but particularly in the months leading to the monsoon where power goes for up to 16 hours a day; and difficulties in the transportation of equipment and individuals (both because of the terrain, and the broader infrastructural developments, like poorly maintained roads, washed away in the monsoon). Poor Internet connections, limited access to scientific journals, limited opportunities for attendance at scientific conferences and debates due to lack of financial resources as well as strict visa regulation in the high income countries, all contribute to the exclusion from access to the global circulation of resources. While individual local researchers are high on demand to work as consultants, and often oversubscribed with multiple research engagements, many have successfully developed their capacity to move between different organizations within or outside of the country or are poached by them. All these issues limit the capacity for sustained development of institutional research capacity within Nepal.

## Emerging regulatory and ethical field

We turn now to the regulation of this emergent field of research practices. Prior to 1991, the Government of Nepal monitored health related research through the Nepal Medical Research Committee based in the Ministry of Health, which was established on 15 April 1982. Many externally funded or sponsored research projects, often with Nepali researchers working as junior research partners or consultants, obtained research and ethical clearance from their own review bodies in the Northern countries, not obtaining permission from Nepal at all. What local reviews were done up to this time was conducted under the chairmanship of the Secretary of Health. The sweeping political changes that followed in the wake of the revolution in 1990 in Nepal leading to the reinstatement of democratic process^23^ , also resulted in changes within state run institutions. The NHRC was set up in 1991, by an act of parliament, with the mandate to, on the one hand, promote research culture, and on the other, review, regulate and approve research proposals in relation to health. Its budget comes from the Ministry of Health. The building in which the NHRC is housed sits behind the Ministry of Health, and access is through the same main entrance way, where visitors need to be signed in by police guards. The executive board of the NHRC is politically appointed. Seven of them are nominated by the Cabinet and five are representatives from different institutions including the Ministry of Health and Population, the Ministry of Finance, the National Planning Commission, Chief of the Research Committee of the Institute of Medicine and the Chairperson of the Nepal Medical Council. The executive chief of the council can either be the chairperson or the member secretary.

From 1991, review of health related research was carried out through the Scientific and Ethics Committee at the NHRC. In addition, further formalization of approach resulted in the first National Guidelines for Ethical Review, published in 1995; these were formally approved by the Ethics Board of the NHRC in 2001; and the Ethical Review Board (ERB) in its current form was established in that same year. The Executive Committee of the NHRC nominates the ERB. In June 2012 the constitution of the ERB was as follows: eleven members in total, with a gender balance of nine men to two women, and almost entirely made up of biomedical doctors (n=9), reflecting this particular disciplinary bias. Among these there were a pediatrician, a public health specialist, obstetrician/ gynecologist, surgeon, general physician, dental surgeon, Ayurvedic physician, lawyer, and a biostatistician. A team of research officers in the research office manages the administration of applications and the review process.

Any new health related research conducted in Nepal has to be submitted to the NHRC following the format available on their website.^24^ The parameters for what constitutes research into health remain somewhat unclear, however, with policy oriented and health systems research not explicitly fitting into this category. For example, researchers and managers of programmatic interventions interviewed often had a question: does behavioral research into Maternal and Child Health issues fall under the rubric of development, or more medically defined bio‐medical health? While there has been an increase in the studies seeking ethical approval, there is no uniform understanding regarding what kind of research studies needs to get NHRC ethical approval. Informants from a few institutions maintained that only studies that have the component of biological samples should take ethical approval although the NHRC maintains that all research in the health sector, regardless of their type, need to undergo their process of ethical review. One research manager of a major research organization told us that it is mandatory to take approval for studies taking biological samples but they did not take approval for behavioural studies.

Thus seeking ethical approval from the NHRC seems to depend on the initiative and the understanding of the concerned investigators. NHRC officials we interviewed maintain that there is large number of research activities taking place within the framework of programmatic intervention that are not always submitted for ethical review. Critics, on the other hand, argued that NHRC interest on ethical review of everything that is concerned with the generation of evidence is primarily driven by its “rent seeking behavior” in that it charges 3% of the total research budget for research projects costing more than $10,000 and $100 for those costing less than $10,000.

The basic guiding principles of review were presented by the Coordinator of the ERB at the workshop on ethics we convened as follows: That the proposed research is essential; that human participation is voluntary; that research on children is conducted only if there is a potential benefit to child's welfare (and only with parental consent, and the agreement of the child); that research on pregnant women is performed only if it contributes to their welfare; and that vulnerable groups are protected by appropriate mechanisms. He presented the basic bioethical principles, of non‐maleficence and beneficence; of the need for appropriate compensation; that the researchers are competent; and that the distribution of both burden and benefits for the research have been carefully thought through.

In addition, he stated the research also needs to address the following: That mechanisms for dissemination of research findings exist; that the research has good institutional support; that mechanisms for maintaining the confidentiality of data exist; that researchers, sponsors and funding agencies accept the legal responsibility for the research. The final guiding principles make up the list: That conflict of interests are declared and are transparent; that externally funded research should resonate with the needs of Nepal; and that the transport of any biological materials out of the country should only be for the purpose stated in the original proposal. In order to facilitate the work of the ERB, a Standard Operating Procedure (SOP) has been developed. However, NHRC policy and regulations neither define health systems research nor do they have any provision on it.

Our research showed that over the years the review process at the NHRC has improved significantly. It has a system in place for carrying out reviews. After the study has been reviewed by the internal reviewer of the council, it is sent to an external reviewer. As of 2012, a reviewer was paid a remuneration of Nepali Rupees 1,200 ($15). The NHRC maintains a roster of experts for reviewing the research proposals. There are certain checklists on relevance, methods, variables etc. that are looked at while reviewing the proposals. The comments or questions from the reviewer are sent to the researchers and the responses from the researchers are sent back to the reviewer after which the proposal, if deemed appropriate, is given approval. Care is taken around conflict of interests when assigning proposals to review.

There are varied perceptions regarding how the NHRC functions. One of the major critiques of the organization is the time taken. While some of the organizations maintain that there has been a change in the scenario from the past where the council used to take up to three months to review a proposal, they feel that the NHRC still lacks the capacity and human resources to undertake its activities in a timely manner. An administrative officer at the NHRC told us that they do not have enough human resources to monitor studies. It uses a part of the application fee to support monitoring of research studies. Being dominated by medical professionals, the NHRC also lacks capacity to review the varied kinds of research proposal in the health sector and thus draws on expertise of independent consultants for reviews.

Given that the NHRC charges 3% of the total budget of a research study for research projects costing more than $10,000, as a fee to review the research proposal, there is dissent amongst the local and international researchers regarding this uncapped fee structure. Some research organizations feel that the fee should be capped or they end up paying a huge amount of money for large research projects. In collaborative research projects, often there are no allocated funds for this purpose, which could create problems to conduct the research projects in Nepal. This issue of uncapped fee has remained unresolved.

Finally, the NHRC does not have the capacity to review increasing number of applications all by itself. It has delegated responsibility to other research oriented entities. In particular, the increasing number of medical and nursing schools and the research activities within these medical educational institutions has led to the establishment of institutional review committees (IRCs). As of 2012, 20 medical education institutions have been given approval to run IRCs. Institutions are allowed to run their own IRCs, but they should be established according to the guidelines of the NHRC.^25^ These need to be approved by the ERB of the NHRC, and report every six months on their activities. The IRCs are allowed to review and approve studies that are carried out by students as a part of their degree or internally funded research. For research at national or international levels, multi‐sited research, externally funded research and clinical trials, they have to seek approval from the ERB itself. Apart from the guidelines, there is very little understanding, however, on how these IRCs function and review the proposals.

## Concluding remarks: NHRC policy in the context of Nepal's political economy of research

What are the implications of this political economy of health sector research on the formation of NHRC guidelines, policy and practice? How are the emerging regulatory and ethical fields taking into account these complexities?

*First,* the Nepal case shows that there is a tension between a definition of what counts as health research and therefore should be subjected to ethical review by the NHRC, and what does not count as health research or counts merely as quality improvement, monitoring and evaluation exercises. While this is not unique to Nepal, this becomes particularly important when most health research activities in the country are organised as programmatic interventions and in the form of studies examining feasibility and efficacy of a particular model of service delivery, monitoring and evaluation, programme reviews and assessments etc. A first step, therefore, is to develop a census of health research activities through mapping, which could give a more accurate sense of the overall research activities.

*Second*, due to limited resources and that the NHRC board's term is shaped by frequent changes in the government, ethical review at the NHRC takes longer than expected. There is not enough expertise available at NHRC for different types of research. For short‐term research projects, there is not much time to get the review process sorted, as this is not usually built in into the timeline proposed by the sponsors. Moreover, the availability of the ERB members could also be an issue, as the meeting of the ERB needs to take place with the presence of at least 6 out of 11 members, for the approval of the study.

*Third*, given that many research studies tend to have foreign investigators, the NHRC has made a mandatory policy that there should be a Nepali Co‐Investigator (Co‐I) in the application. While NHRC policy is informed by an approach to enhance local capacity, these enforced mechanisms do not always lead to active participation of Co‐Is, who may be included mainly to meet the policy requirement rather than as genuine research collaborators leading to enhancing research culture. Therefore, it might be useful to think of a broader approach in terms of encouraging Co‐Is to have protected time for research as well as including participation in research and publication as criteria for their promotion and career progression.

*Fourth*, the nature of research collaborations has often increased individual capacity at the expense of institutional and organizational research capacity (as reflected in intra organizational transfer of researchers, and poaching). The unequal relationship between the sponsors and the sub‐contractors mean that sub‐contractors have very little control over both the overall design and what happens to the research results once they are submitted. Although the local research organizations as sub‐contractors end up doing most of the work on the ground, they rarely received direct funding from sponsors. There is very little resources available to meet indirect costs for carrying out research activities. This has implications for the limited ownership of data, and indirect sub‐contracted budgets allows for little or no protected time to work on research publications.

*Fifth*, responsibility for the publication of research results remains unclear. Results are either published in international journals–where there is now frequently provision for cheaper open access if there are low‐income country authors–or are unknown, and unpublished. A provision of the terms for permission to undertake research could be insistence on publication in Nepalese journals in addition to open‐access international journals and other publication outlets.

In conclusion, one of the key challenges in global health is the lack of research capacity in low and middle‐income countries. The Director General of the WHO suggested in 1998 that improving health and reducing poverty in developing countries requires a quantum leap in capacity building’.^26^ There has been a perceived change in the position of Nepali research institutions, which previously mainly worked to implement pre‐designed research and acted as data couriers to Northern partners. Despite many now being involved as research partners, there is very little evidence that such a shift is changing the rules of the game. The unequal field in which contract research and international research collaboration takes place in low‐income countries raises questions that need to be answered from a broader perspective than that which can be addressed from within the ethics board. Moreover, an increasing number of research activities in low‐income countries that are organized around experimental programmatic interventions. These are often sponsored and conducted by international collaborative assemblages of aid agencies, policy makers, NGOs and research organisations. Their direct influence in shaping policies and programmes calls for greater public accountability and a broader definition of what constitutes the ethics of health and development interventions.

## Notes

1 C. G. Victora, J. P. Habicht & J. Bryce. Evidence‐based public health: moving beyond randomized trials. *Am J Public Health* 2004; 94: 400–405; V. Li, S. M. Carter & L. Rychetnik. Evidence valued and used by health promotion practitioners. *Health Educ. Res*. 2015; 30: 193‐205; S. Lifsey et. al. Building the evidence base for population‐level interventions: barriers and opportunities. *Health Educ Behav*. 2015; 42:133S‐140S.

2 C. McMichael, E. Waters & J. Volmink. Evidence‐based public health: what does it offer developing countries? *J Public Health (Oxf)* 2005; 27: 215‐221.

3 A. Petryna. 2009. *When Experiments Travel: Clinical Trials and the Global Search for Human Subject*. Princeton. Princeton University Press.

4 M. Boulton & M. Parker. Informed consent in a changing environment. *Soc Sci Med*. 2007; 65: 2187‐98; I. Harper. Translating ethics: Researching public health and medical practices in Nepal. *Soc Sci Med*. 2007; 2235‐2247.

5 A. A. Hyder et. al. Ethical review of health systems research in low‐ and middle‐income countries: a conceptual exploration. *Am J. Bioeth*. 2014; 14: 28‐37.

6 R. Macklin. 2004. *Double Standards in Medical Research in Developing Countries*. New York; Cambridge University Press.

7 Presentations were made by a representative of the BHESA team, a Coordinator of the Ethical Review Board (ERB) at NHRC, the Director of a Nepal based bio‐medical research center and a Professor of a private medical college based in Kathmandu who was also the former secretary of the Institutional Review Committee (IRC) of the same institution. There were a total of 13 participants in the workshop.

8 Dr Carl E. Taylor from the Chicago Natural History Museum expedition carried out Nepal's first health survey in Kali Gandaki region in central Nepal in 1949.

9 J. M. Isaacson et. al. 2001. *Half‐a‐Century of Development: The History of U.S. Assistance to Nepal, 1951‐2001*. Kathmandu, Nepal. USAID: 43.

10 R. M. Worth & N. K. Shah. 1969. *Nepal Health Survey, 1965‐1966*. Honolulu, University of Hawaii Press.

11 Isaacson et. al., *op. cit*. note 9, p. 90.

12 J. Justice. 1986. *Policies, Plans and People: Culture and Health Development in Nepal,* Berkeley, University of California Press: 112.

13 I. Harper. 2014. *Development and Public Health in the Himalaya: Reflections on healing in Contemporary Nepal*. London and New York. Routledge; J. Justice. 1986. *Policies, Plans and People: Culture and Health Development in Nepal,* Berkeley, University of California Press.

14 K. P. West et al. Efficacy of vitamin A in reducing preschool child mortality in Nepal. *Lancet* 1991; 338: 67–71.

15 N. M. Daulaire et. al. Childhood mortality after a high dose of vitamin A in a high risk population. *BMJ* 1992; 304: 207–210.

16 I. Harper. Capsular promise as public health: A critique of the Nepal National Vitamin A Programme. *Studies in Nepali History and Society* 2002; 7: 137‐173.

17 M. R. Pandey et. al. Reduction in Total Under‐five Mortality in Western Nepal through Community‐based Antimicrobial Treatment of Pneumonia. *Lancet* 1991; 338: 993–97.

18 P. Dawson et. al. From research to national expansion: 20 years' experience of community‐based management of childhood pneumonia in Nepal. *Bull World Health Organ* 2008; 86: 339–43.

19 Determination of what counted as health experiments was a difficult task for us, mainly because there was no central database that categorized health research activities as experimental or non‐experimental. Our definition of what counted as health experiments and therefore fitted our inclusion criteria included: studies that were explicitly labeled as experiments, quasi‐experiments, efficacy studies, intervention studies, diagnostic tests and impact/evaluation studies.

20 This trial in Nepal had a controversial history: the Hepatitis E vaccine trial sponsored by the US Army as a Phase II/III clinical trial came under sustained criticism. Community and political leaders raised objections concerning the US army's interest in vaccines for a disease found in Nepal, and how the trial participants and other local people would benefit from the research. For details, see: Harper, *op cit*., note 4.

21 For details on the rise of private medical institutions and nursing schools, see: I. Harper. 2011. World Health and Nepal: Producing Internationals, Healthy Citizenship and the Cosmopolitan. In David Mosse (ed) *Adventures in Aidland: The Anthropology of Professionals in International Development*. Berghahn: 123‐138; R. Adhikari. 2008. The Business Nursing Complex: Understanding Nursing Training in Nepal. *Studies in Nepali History and Society* 13; 297‐322.

22 B. Simpson et. al. Pharmaceuticalisation and ethical review in South Asia: Issues of scope and authority for practitioners and policy makers. *Soc Sci Med* 2015; 131: 247‐254; S. Sariola et. al. Big‐pharmaceuticalisation: Clinical trials and Contract Research Organisations in India. *Soc Sci Med* 2015; 131: 239‐246.

23 J. Whelpton. 2005. *A History of Nepal*. Cambridge: Cambridge University Press; M. Hofton, W. Raeper & J. Whelpton. 1999. *People, Politics and Ideology: Democracy and Social Change in Nepal*, Kathmandu: Mandala Book Point.

24
**For all applicants:**1. Cover letter addressing to Member Secretary indicating that submission of proposal for Ethical Approval; 2.Submission of the protocol in the NHRC format; 3.Photo of PI/ co‐ PI; 4.Applicant's Signature; 5.Curriculum vitae of the Principal Investigator, Co PI and other personnel who are involved in the study; 6.Submission of the application processing fee as per the rules and regulations of NHRC; 7 Bibliography and references of the research study; 8.List of Abbreviations/ acronyms; 9. If the research study is to be conducted in any hospital or institution acceptance letter from the related hospital or the institution; 10; Consent form should be in Nepali & local language (if necessary); 11, Electronic copy of the proposal in CD; 12. Tools and guidelines for data collection in Nepali; 13. Consent form in Nepali if conducted in Nepal.

25 Nepal Health Research Council (NHRC). 2011. *National Ethical Guidelines for Health Research in Nepal and Standard Operating Procedures*. Kathmandu: NHRC.

26 T C. Nchinda. Research capacity strengthening in the South. *Soc Sci Med*. 2002; 54: 1699‐711.

